# Potential Hepatotoxins Found in Herbal Medicinal Products: A Systematic Review

**DOI:** 10.3390/ijms21145011

**Published:** 2020-07-16

**Authors:** Nguyen Van Quan, Tran Dang Xuan, Rolf Teschke

**Affiliations:** 1 Transdisciplinary Science and Engineering Program, Graduate School of Advanced Science and Engineering, Hiroshima University, Hiroshima 739-8529, Japan; nvquan@hiroshima-u.ac.jp (N.V.Q.); tdxuan@hiroshima-u.ac.jp (T.D.X.); 2 Department of Internal Medicine II, Division of Gastroenterology and Hepatology, Klinikum Hanau, Teaching Hospital of the Medical Faculty, Goethe University Frankfurt/Main, 63450 Hanau, Germany

**Keywords:** contaminants, hepatotoxicity, herbal medicinal products, liver injury, herb induced liver injury, RUCAM, nonphyto-hepatotoxins, phyto-hepatotoxins

## Abstract

The risk of liver injury associated with the use of herbal medicinal products (HMPs) is well known among physicians caring for patients under a HMP therapy, as documented in case reports or case series and evidenced by using the Roussel Uclaf Causality Assessment Method (RUCAM) to verify a causal relationship. In many cases, however, the quality of HMPs has rarely been considered regarding potential culprits such as contaminants and toxins possibly incriminated as causes for the liver injury. This review aims to comprehensively assemble details of tentative hepatotoxic contaminants and toxins found in HMPs. Based on the origin, harmful agents may be divided according two main sources, namely the phyto-hepatotoxin and the nonphyto-hepatotoxin groups. More specifically, phyto-hepatotoxins are phytochemicals or their metabolites naturally produced by plants or internally in response to plant stress conditions. In contrast, nonphyto-hepatotoxic elements may include contaminants or adulterants occurring during collection, processing and production, are the result of accumulation of toxic heavy metals by the plant itself due to soil pollutions, or represent mycotoxins, herbicidal and pesticidal residues. The phyto-hepatotoxins detected in HMPs are classified into eight major groups consisting of volatile compounds, phytotoxic proteins, glycosides, terpenoid lactones, terpenoids, alkaloids, anthraquinones, and phenolic acids. Nonphyto-hepatotoxins including metals, mycotoxins, and pesticidal and herbicidal residues and tentative mechanisms of toxicity are discussed. In conclusion, although a variety of potential toxic substances may enter the human body through HMP use, the ability of these toxins to trigger human liver injury remains largely unclear.

## 1. Introduction

In recent years, the topic of convincing efficacy observed along with the use of herbal medicinal products (HMPs) and the associated risk of liver injury has been of major concern among scientists, physicians, pharmacists, manufacturers, and regulators [1,2,3,4,5,6]. In the context of toxicity, the question remained regarding the source of poisons, either associated with the plant itself or related to exogenous poisons. It is undeniable that the use of HMPs has not only brought some remarkable results in medical treatment but also led to great achievements in science and economics [1]. Traditional medicines are a potential treasure derived from indigenous knowledge of using natural products to treat human diseases or improve at least minor ailments. Since ancient times, raw plants as well as refined plant products are in common use, of which, traditional Chinese medicine (TCM), Ayurveda in India, Kampo in Japan, traditional Korean medicine, and Unani in old Greece are the most outstanding ones. Recently, the discovery of new compounds from plants that can substantially contribute to alleviating several serious widespread diseases in the world merit mentioning, such as the discovery of artemisinin by Youyou Tu for therapy of malaria, and not to forget William Campbell and Satoshi Ōmura who disclosed avermectins for the treatment of helminthic diseases [1]. On the other hand, some modern therapies have been successful in concomitant use with herbal medicines [3]. However and despite of anecdotal evidence and widespread use in several countries, well-conducted randomized controlled trials (RCTs) supporting the efficacy of HMPs are largely lacking. Thus, the efficacy of herbal remedies is still unclear. Moreover, there is concern regarding the prevalent use of HMPs since the risk of use may be too high because of reported liver injury [1,3,4,5,6]. Most hepatotoxic injuries associated with the use of HMPs have been reported from herbal TCM with clear evidence for a causal relationship [3,7,8]. Although main hepatotoxic TCM plants and their ingredients have been investigated, groups of hepatotoxic phytocompounds and contaminants from other HMPs have not been adequately congregated.

In this systematic review, we classified two groups of hepatotoxic compounds and contaminants including hepatotoxic phytochemicals and nonphyto-hepatotoxins found in HMPs. The MEDLINE/PubMed database was used to follow the guidelines of Preferred Reporting Items for Systematic Reviews and Meta-Analyses (PRISMA) and searched for key terms.

## 2. Methodology and Search Strategy

**Figure 1:**
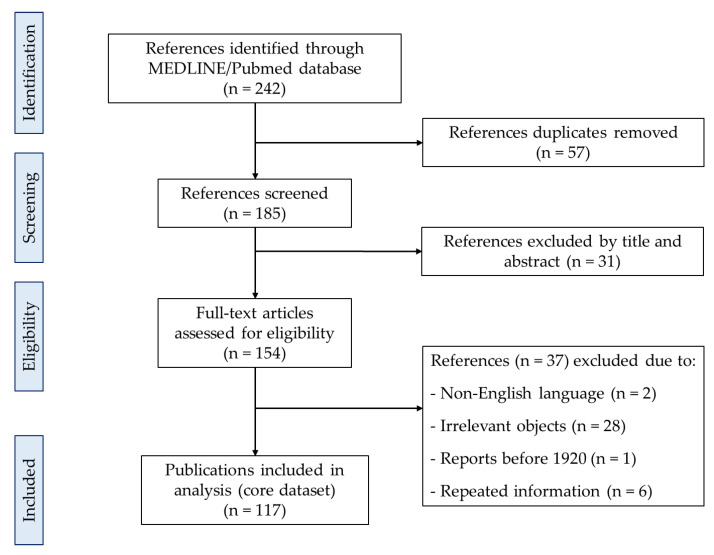
PRISMA flow diagram of references included in the review.

The principles of PRISMA were used for this systematic review article (Figure 1). For literature search, the search terms included hepatotoxic medicinal plants; herbal medicine induced liver injury; hepatotoxic natural compounds; phytochemicals induced liver injuries in order to preliminarily determine the medicinal plant species and toxic components. The targeted compounds and contaminants were then searched for together with each of the terms hepatotoxicity, liver injury, and liver failure to confirm the toxic property by using the same database. A total of 242 scientific publications were screened and filtered. Eventually, 117 publications were selected as the core dataset, all of which were cited in this review. The selective search was based principally on English language reports from 1920 to May 30, 2020 by reputable publishers.

## 3. Phyto-Hepatotoxins from HMPs

The term “phyto-hepatotoxins” in this review implies any potential hepatotoxic compound synthesized by medicinal plants, which may be regulated by the plant’s intrinsic genomics or be influenced by extrinsic factors during plant’s life. A number of phytochemicals have been identified with benefits for human health including hepatoprotective effects [9], but more liver injury cases induced by phytocompounds have been reported with increasing frequency. A list of potentially hepatotoxic compounds found in some commonly used HMPs is provided for a quick overview with added references that also proposed indications of treatment, although therapeutic efficacy has rarely been verified by RCTs (Table 1). 

Eight main phytochemical groups with potential hepatotoxicity are listed (Table 1) with focus on volatiles compounds, phytotoxic proteins, glycosides, terpenoid lactones, terpenoids, alkaloids, anthraquinones, phenolic acids. Most of them are secondary metabolites produced with the aim to protect medicinal plants (MPs) from outside attacks to allow a longstanding plant survival. Apparently, secondary compounds of HMPs are cornerstones for known or assumed medicinal and pharmaceutical properties. Nevertheless, besides the potential beneficial effects, the parallel existence of toxic compounds in such products may induce unexpected adverse effects. Recently, overall 296 phytochemicals have been reported causing liver injury, of which alkaloids and terpenoids are the two major groups of hepatotoxicity [104]. In the present analysis, besides those two groups, we found that some representatives of volatile compounds, proteins, glycosides, saponins, glycoside-saponin compounds, terpenoid lactones, anthraquinones, or even several phenolic compounds also possess the potential hepatotoxicity (Table 1).

The toxicity of volatile compounds from most traditional herbal medicines is commonly believed to vanish by degrading due to high temperatures during the decoction preparation process, conditions that do not apply to pennyroyal oil, which has a long historical use as a dangerous abortifacient and as a means to induce menstruation, with pulegone as its major compound to be classified as a strong hepatotoxin [11,12]. Instead, the number of toxic proteins from commonly used MPs is likely low.

Glycosides, saponins, and glycoside-saponin compounds have increasingly been viewed as toxic chemicals in reports on liver toxicity. Kaurene, atractyloside, carboxyatractyloside, and 4′-desulphate-atractyloside are known poisonous glycosides causing liver injury, found in medicinal plants such as *Xanthium strumarium* and *Callilepis laureola* [20,21,22,23,24]. Besides, cycasin from cycad plants was early recognized to cause experimental hepatic injuries such as toxic hepatitis and cirrhosis in nonhuman primates [25,26]. Saponin compounds and their derivatives have been linked to toxic hepatitis and were identified in various MP species consisting of *Albizia julibrissin*, *Dumasia truncata*, *Anemarrhena asphodeloides*, *Melia azedarach*, *Tripterygium wilfordii* [22,23,24,25,26,27,28,29,30,31,32,33,34,35]. In this context, monodesmosyl saponin 3-*O*-*α*-l-rhamnopyranosyl-(1→3)-*β*-d-glucuronopyranosyl-28-*O*-*β*-d-glucopyranosyl oleanolic acid from *Dumasia truncata* showed the most remarkable toxicity toward liver cells [29].

Hepatotoxic effect of terpenoids and terpenoid lactones has been reported in association with the use of several MPs. Among toxic terpenoids, triptolide from *Tripterygium wilfordii*, toosendanin from *Melia toosendan*, lantadenes A and B from *Lanata camra* are the most frequently reported compounds capable of inducing liver injury. By in vitro and in vivo assays, terpenoid lactones found in *Dioscorea bulbifera*, *Helenium aromaticum*, *Telekia speciosa*, *Aucklandia lappa*, *and Inula helenium* have been shown to be involved in the stimulation of glutathione peroxidase activity and reduction of glutathione levels which in turn may trigger the initiation and progress of liver injury [43,44,45,46,47,48,49,50,51,52,53]. 

The most potent hepatotoxic anthraquinones have been identified in two species of *Polygonum* genus, namely *P. multiflorum* and *P. cuspidatum*. The toxicity of this compound group is seemingly associated with the suppression of bile acids transporters and activation of mitochondrial apoptosis pathway [104]. In addition, the hepatocyte apoptosis caused by anthraquinones has been linked to oxidative stress [105]. 

Switching from toxic to more beneficial ingredients, phenolic compounds and phyto-metabolites with phenolic-based structure have commonly been assumed as beneficial for human health, although liver injuries by phenolics have rarely been reported. As an example, phenolic acids and catechins containing gallic acid moieties from green tea *Camellia sinensis* are viewed as hepatotoxins, if derived from green tea extracts, and via the mechanisms of mitochondrial membrane potential collapse and reactive oxygen species (ROS) formation [90]. The causality assessment used the Roussel Uclaf Causality Assessment Method (RUCAM) as outlined in our previous reports, confirming increased risks of liver injury after consumption of green tea extracts [92,106]. Among other MPs, the hepatotoxicity of eugenol, methyleugenol, and acetyleugenol have been well documented [104]. The major mechanism of their toxicity is attributed to reduced hepatic glutathione levels using experimental mouse models. Other phyto-hepatotoxic compounds containing benzene ring have been identified in *Larrea tridentata*, *Sassafras albidum*, and *Guatteria gaumeri* comprising nordihydroguaiaretic acid, safrole, and α-asarone, respectively.

**Figure 2:**
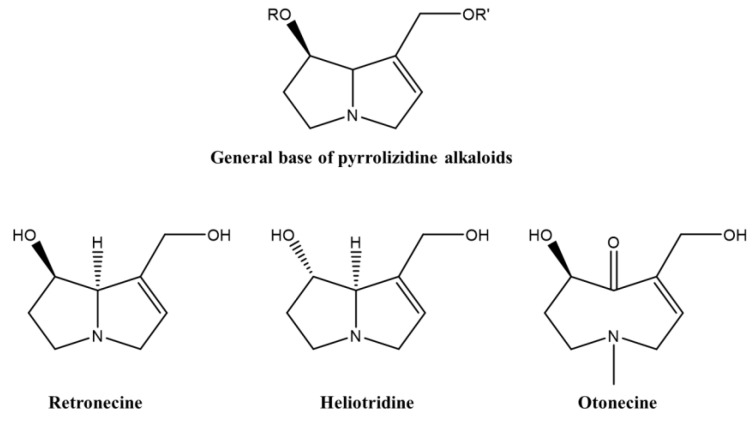
General structure of pyrrolizidine alkaloids.

Among the many hepatotoxic substances, the pyrrolizidine alkaloids (PAs) have been thoroughly scrutinized regarding mechanistic steps and clinical consequences. Interestingly, hepatotoxic PAs are found in various MPs used as medicine. Among these is comfrey (*Symphytum* species), a PA containing herbal plant that is used for the treatment of inflammatory conditions such gout, arthritis, thrombophlebitis, skin wounds (bruises, fungal infections, ulcers, fractures, strains), varicose veins, oral lesions (gargle), bronchitis, allergies, gastritis, gastroduodenal ulcers, and diarrhea in Western Europe [59]. The hepatic problems induced by PAs were first revealed in 1920 by a study of Wilmot and Robertson, who described liver cirrhosis as a senecio disease associated with the ingestion of *Senecio* species in South Africa [107]. To date, a diverse number of PA-induced liver injuries has been reported, in which the most toxic PAs are identified as macrocyclic diesters of retronecine, heliotridine, and otonecine (Figure 2) [108].

**Figure 3:**
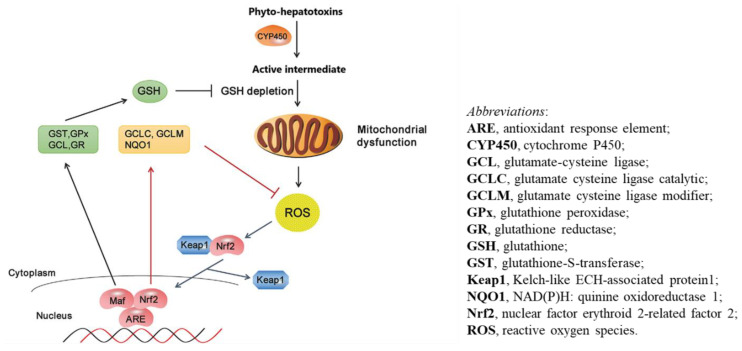
Phyto-hepatotoxins induce liver injury through oxidative stress. The mechanic pathway is derived from a published report by Xu et al. [109].

**Figure 4:**
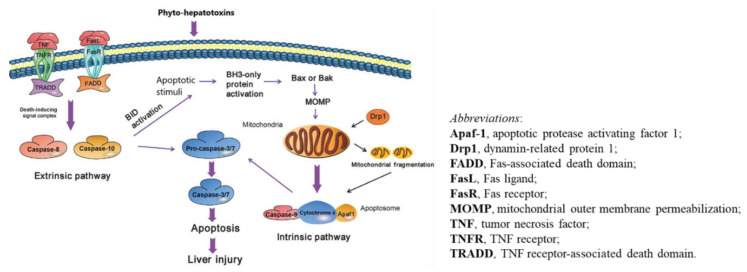
Phyto-hepatotoxins induce extrinsic (death receptor) and intrinsic (mitochondrial) apoptotic pathways. The pathways are adapted from the report of Xu et al. [109].

The general mechanism of PAs and other phytocompounds inducing liver injury can be explained by oxidative stress, apoptosis, and abnormality of bile acid metabolism pathways (Figure 3 and Figure 4). Most of these hypotheses were derived from experimental studies using animal models simulating liver injury by PAs in patients who used herbs containing PAs and experienced hepatic sinusoidal obstruction syndrome (HSOS).

Within the oxidative stress mechanism, phyto-hepatotoxins are first metabolized by CYP450 and generate an active intermediate, which diminish hepatic GSH levels and cause mitochondrial dysfunction, resulting in overproduction of ROS (Figure 3). Subsequently, ROS leads to activation of signaling events including Nrf2-mediated activation of genes containing antioxidant response element (ARE). Nrf2 nuclear translocation and the Nrf2-mediated antioxidant defense system contribute to preventing excessive ROS levels from accumulating at the cellular and tissue levels [109].

The mechanism of hepatocyte apoptosis relating to phyto-hepatotoxins is outlined graphically and is still hypothetical (Figure 4). In short, apoptosis results from the activation of either death executioner caspase-3 or -7 which is the consequence of a chain of complex signaling transmission and metabolism by both extrinsic (by death-inducing signal complex) and intrinsic (by mitochondrial dysfunction) pathways through an agent factor like phyto-hepatotoxins.

On the other consideration, bile acids are indispensable signaling molecules, playing an important role in regulating its own homoeostasis as well as adjusting cholesterol and lipid homoeostasis [110]. The imbalance or dysfunction of bile acid content in the body may lead to hepatic problems. Previous studies found an obvious evidence between the usage of MPs containing PAs and liver injuries via the impaired metabolism of bile acids [109]. Particularly, increased serum bile acids could be a sensitive index of hepatic function caused by PAs [111]. Xiong et al. [112] found that the detoxification process of senecionine as a typical macrocyclic PA was obstructed leading to enhanced hepatic bile acids accumulation and caused injury. On the other hand, bile acids are also toxic to liver cells, and their regulation is occasionally considered as a sensitive biomarker for assessing some forms of liver injury. Therefore, further studies on other phyto-hepatotoxins associated with bile acids homeostasis should be implemented to clarify the unknown mechanism of HMPs-induced hepatotoxicity.

Clearly, liver injury by herbal products can be caused by toxic phytochemicals (Table 1) to be differentiated from those chemicals that are contaminants rather than ingredients of these products, representing nonphyto-hepatotoxins.

## 4. Nonphyto-Hepatotoxins from HMPs

The term “nonphyto-hepatotoxins” stands for substances and agents thatinduce liver injury and are contaminated to HMPs but not synthesized by MPs. The impurity of HMPs can be caused by biotic and/or abiotic factors with the possible contamination of dust, pollens, insects, rodents, parasites, microbes, fungi, moulds, toxins, heavy metals, pesticides, herbicides and undeclared chemicals [7,113,114]. Based on published reports we divide these contaminants into three main groups including metals, mycotoxins, as well as pesticidal and herbicidal residues (Table 2).

Metals are available in soil and water, therefore they can be absorbed by and accumulated inplants through the nutrient metabolism process. As a consequence, medicinal plants grown in a place with high concentration of metals may have a high risk of metal contamination and accumulation. Besides, HMPs can be polluted with toxic metals through pesticide and herbicide products as well as via transportation and preservation processes. The most common toxic metals detected in HMPs are arsenic, mercury, cadmium, nickel and lead [115,116]. Early studies indicated the poisonous impact of heavy metals overload on the human liver, which initiates the pathogenesis of oxidative stress to hepatocytes, metabolism dysfunction, and eventually liver injury [115,117]. Later studies have observed the similar phenomenon [1,7,118].

HMPs are also poisoned by biological factors including bacteria, fungi, yeast, and parasites with their spores and some toxins, which can easily cause misunderstandings about the actual advantage of some medicinal herbs. Among reported toxic elements, mycotoxins, a group of toxic secondary metabolites, which are primarily produced by *Aspergillus*, *Penicillium*, *Fusarium*, *Claviceps*, and *Alternaria* fungi. The mycotoxins most damaging the human health are aflatoxins, ochratoxin A, fumonisins, zearalenone, and deoxynivalenol [121]. Contamination with these poisons has been recognized in various HMPs during their agricultural and manufacturing processes. The hepatotoxic effects of mycotoxin mixtures were proved comprising hepatic total antioxidant capacity decrease, lipid peroxidation increase, and potential hepatic apoptosis by upregulation of the apoptotic genes Caspase-3 and Bax [120]. In the case of kava, *Piper methysticum*, aflatoxins contaminated the medicinal plant due to hot and humid weather conditions are considered potential culprits of kava hepatotoxicity [78,122,132].

The third group of contaminants in HMPs relate to pesticidal and herbicidal residues. It is recognized that the superior effects of synthetic herbicides and pesticides areon crop protection and agricultural production. However, overuse of these synthetic substances may have a long influence on environmental pollution and human health issues. The reported liver effects of pesticide and herbicide residues include hepatocellular hypertrophy and cytoplasm degeneration, obstructive cholestasis, decreased acetylcholinesterase activity, and hepatic mitochondrial toxicity [114,123,124,125,126,127,128,129,130,131].

**Figure 5:**
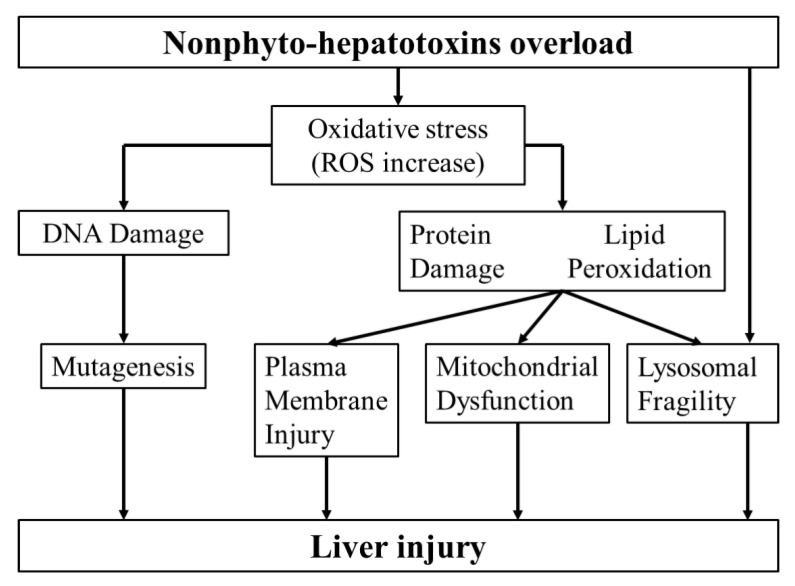
Summarized hypothetical mechanisms of liver injury induced by nonphyto-hepatotoxins overload. Modified from a report published by Britton [115].

A condensed hypothesis of mechanisms leading to liver injury by nonphyto-hepatotoxins is illustrated (Figure 5). Accordingly, the absorption of nonphyto-hepatotoxins by the liver may lead to increased ROS production and lysosomal fragility. Under the oxidative stress, macromolecules as proteins and DNA in hepatocytes are damaged and lipid peroxidation processes rise. The dysfunction of subcellular organelles including lysosomes and changes of hepatocytes with their mitochondria and plasma membranes are considered of particular relevance in causing liver injury (Figure 5).

## 5. Critical Analysis and Reality Check

Liver injury by herbs [133,134] and drugs [135] can best be assessed for causality by RUCAM [136,137]. HILI in patients consuming herbal products is a particular clinical challenge as evidenced by the large number of HILI cases [133,134] published using RUCAM for causality assessment with details provided for worldwide published 12,068 HILI cases [133] that included 11,160 HILI cases published by authors from Asia [134]. These figures were much lower compared with those of the RUCAM based 46,266 DILI cases published from 2014 up to early 2019 [135]. The analyses of RUCAM based HILI and DILI cases revealed that most case data were of good quality ready for appropriate causality assessment [133,134,135]. Authors from around the world commonly used RUCAM for their cases smoothly and without discussing any problems during its application. In a few HILI cases presented in an extra list, causality for several herbs, initially implicated in suspected HILI by regulatory agencies from Germany or the US, could not be verified using RUCAM [133,134]. Among the RUCAM based DILI cases, most received a probable or highly probable causality level [135]. However, published were also DILI cases with a possible or excluded causality grading for the implicated drug, mostly due to incomplete data or if cohorts were assessed retrospectively not allowing for prospective collection of complete important data relevant for assessment by RUCAM [135], Indeed, high causality gradings are achieved with complete data provided by prospective studies as strongly recommended by the updated RUCAM [137].

RUCAM in its original version published in 1993 [136] and as update reported in 2016 [137] represent sophisticated diagnostic algorithms [136,137,138,139] based on principles of artificial intelligence (AI) as outlined in a recent editorial [140]. In more detail, AI principles recommend the use of algorithms for solving complex processes, done for the complex multifaceted DILI and HILI by constructing RUCAM as an intelligent algorithm [136,137]. In order to simplify complex processes, the principle was to provide tools enabling input of data into a black box that systematically evaluates incoming data and fosters output of clear results such as diagnosis in complex diseases. Clearly defined elements of HILI and DILI case features with a scoring system are the bases and components of the intelligent RUCAM algorithm, prepared for receiving case data and providing a final score of probability for any herb or drug implicated in suspected HILI or DILI.

RUCAM is viewed as cornerstone assessing global causality in HILI and DILI cases. Conditions in the context of HILI are more complicated due to confounding variables because causality evaluation is confined to the whole herbal product with all ingredients and does not allow for causality evaluation of individual toxins [133,134]. This provides uncertainty to what extent individual toxins are in reality responsible for the liver injury. A variety of other causative aspects are poorly investigated, among these are adulterations of HM by conventional drugs added by manufacturers to increase product efficacy [113] and not to forget misidentified herbs included in HMPs instead of desired authentic herbs.

Additional concerns focus on the abundant indications as published and proposed for many therapeutic indications that are more than fragile and claimed on the base of data derived from in vitro data preferentially involving antioxidant properties. Finally, the issue remains concerning the efficacy of HMPs for disease treatment, heavily disputed among Western physicians and traditional herbalists as evidence base criteria were not applied in most trials and randomized controlled trials (RCTs) of good quality were rarely published. This obviously leads to the crucial point of the efficacy to risk balance which is negative for most HMPs. Potential hepatotoxic properties of the phyto-hepatotoxins and the nonphyto-hepatotoxins contained in HMPs call for regulatory product standardization and manufactural compound purification of herbal remedies.

In fact, several HMPs have been banned and/or have had warning letters issued by regulatory agencies due to their assumed toxic effects on human health. However, most reports seemingly favor the listing of herbal products associated with various toxicities in general rather than the compounds with hepatotoxicity potency in particular. For instance, the Medicines and Healthcare Products Regulatory Agency of the UK published a list of banned and restricted herbal ingredients [141] but did not mention any specific toxin as well as its adverse effect on liver. Fleischer, Su and Lin indicated that among 300 commonly used Traditional Chinese Medicines (TCMs), Ma Huang (*Ephedra sinica*) was the only herbal product banned by six countries including the USA, the UK, Germany, Israel, Canada and Australia [142]. The Canadian government issued the highest number of banned Chinese herbs (98 species) while the USA was found to have the least restricted ones (9 products) [142]. In the same manner, this report did not provide any information of corresponding hepatotoxins either. Additionally, the easy accessibility of HMPs on internet markets is currently causing difficulty for the surveillance and management by governmental regulators. This suggests that a global standard regulation should be established for more strictly tracking the use of HMPs to avoid the risk of liver injuries by hepatotoxins constituted in HMPs as mentioned in this review. Besides the considerations about accepted amounts of hepatotoxins, the acutely toxic chemicals as ricin, paraquat, or arsenicshould be accurately detected and completely removed from HMPs by advanced techniques with high sensitivity to assure the quality and safety of HMPs.

## 6. Conclusions

HILI cases caused by modern or traditional HMPs including herbal TCMs are challenging if it comes to causality assessment of possible toxins as part of the herbal products. Indeed, there are difficulties assigning toxins as causative agents in most of the published HILI cases. In addition, genetics of individuals make them to patients susceptible to rare liver injury but individual risk factors are poorly assessed. Although a variety of potentially toxic substances may enter the human body through HMP use, the relevance of their property to trigger human liver injury remains unclear in many cases. By this review, potential hepatotoxins can be found in herbal medicinal products with both types of plant originated compounds (phyto-hepatotoxins) and exogenous contaminants (nonphyto-hepatotoxins). Although potential hepatotoxins and their toxicity have been reported, the actual form of liver injury such as acute hepatocellular injury, acute cholestatic injury, mixed injury, sinusoidal obstruction syndrome, vanishing bile duct syndrome, and acute liver failure need to be further elaborated by prospective clinical investigations. Quality evaluation of HMPproducts are urgently required prior to treating patients. The composition and content of potential hepatotoxins included in HMPs must be properly assessed for the risk of liver injury during the treatment. Novel advanced kits that can quickly detect and quantify hepatotoxins in HMPs should be developed with sensors dedicated to organic and inorganic toxins. Moreover, together with the rapid development of digital technology, the combination of RUCAM, artificial intelligence (AI), and available data sources of potential hepatotoxins like those presented in this review may help discover and establish the optimal HMP therapeutics without major risks of liver injury.

## Figures and Tables

**Table 1:** List of phyto-hepatotoxins found in herbal medicinal products

Phyto-Hepatotoxins	Medicinal Plants (Common HMPs)	Claimed Treatment of MPs	References
**Volatile Compounds**			
Volatile oils	*Artemisia argyi* (Ai Ye)	Abdominal pain, dysmenorrhoea, uterine haemorrhage, and inflammation diseases in various countries including China, Japan, and Korea	[3,10]
Pulegone	*Mentha pulegium* *Hedeoma pulegioides* (Pennyroyal oil)	Menstrual discomfort, abortifacient, inflammatory diseases like chronic bronchitis, influenza, and common upper respiratory tract infections	[11,12]
**Phytotoxic Proteins**			
Ricin	*Ricinus communis* (Bi Ma Zi)	Unspecific abdominal disorders, arthritis, backache, muscle aches, schistosomiasis, chronic backache and sciatica, chronic headache, constipation, expulsion of placenta, gallbladder pain, period pain, menstrual cramps, rheumatism, sleeplessness, and insomnia	[13,14]
Abrin	*Abrus precatorius* (Chanothi, Xiang Si Zi)	Neuromuscular disorders, epilepsy, viral infections, malaria, fertility problems, diabetes mellitus, renal insufficiency, inflammatory disorder, and autoimmune diseases	[15,16,17]
Hypoglycins A and B	*Blighia sapida* (Ackee)	Broad range of infections causing fever, dental decay, malaria, internal haemorrhage, dysentery, burns, eye inflammation, yellow fever, constipation, cutaneous infections, whitlow and head lice	[18,19]
**Glycosides**			
Kaurene	*Xanthium strumarium* (Cocklebur, Cang Shan) *Atractylis gummifera* *Callilepis laureola* (Impila, Zulu remedy)	Rhinitis, nasal sinusitis, headache, gastric ulcer, urticaria, rheumatism, arthritis, and bacterial, fungal infections	[20,21,22,23,24]
Atractyloside			
Carboxyatractyloside			
4’-desulphate-atractyloside	*Callilepis laureola* (Impila, Zulu remedy)	Stomach pains and discomfort, constipation, tape worm infections, cough, induction of fertility in young women, and impotence in males	
Cycasin	*Cycas revoluta* (Su Tie)	Cancer including hepatocellular carcinoma, rheumatism, impaired diuresis	[25,26]
Saponins	*Albizia julibrissin* (He Huan Hua, He Huan Pi)	Anxiety, cancer, depression, sleep problems (insomnia), and sore throat; Mood improvement; Reduction of swelling associated with trauma	[22,27]
Monodesmosyl saponin 3-*O*-*α*-l-rhamnopyranosyl-(1→3)-*β*-d-glucuronopyranosyl-28-*O*-*β*-d-glucopyranosyl oleanolic acid	*Dumasia truncata*	Muscle pains	[28,29,30]
Timosaponin	*Anemarrhena asphodeloides* (Zhi Mu)	Common cold-induced febrile disease with arthralgia, hematochezia, tidal fever and night sweats by so called Yin deficiency, bone-steaming, cough, and hemoptoe	[31,32]
Tetranortriterpenoids	*Melia azedarach* (Bakain)	Leprosy, scrofula, rheumatism, ringworm and scabies, malaria fever, and helminthic diseases	[3,33,34]
Multiglycoside	*Tripterygium wilfordii* (Lei Gong Teng - Thunder God Vine)	Rheumatoid arthritis and other immune diseases, autoimmune and inflammatory conditions	[35]
**Terpenoids**			
Triptolide	*Tripterygium wilfordii* (Lei Gong Teng—Thunder God Vine)	Rheumatoid arthritis and other immune diseases, autoimmune and inflammatory conditions	[35,36,37,38]
Toosendanin	*Melia toosendan* (Chuan Lian Zi)	Stomach pains, inflammations, and ascariasis	[39,40,41]
Lantadenes A and B	*Lantana camara* (Latana oil)	Tumor and bacterial infections	[6,42]
**Terpenoid Lactones**			
8-epidiosbulbin E acetate Diosbulbin B Diosbulbin D	*Dioscorea bulbifera* (Huang Yao Zi)	Tumors, cancers and thyroid gland diseases	[43,44,45,46]
Linifolin A Geigerinin Helenalin Mexicanin I	*Helenium aromaticum*	Tumors	[47,48]
6α-Hydroxy-2,3-dihydroaromaticin Asperilin Telekin	*Telekia speciosa*	Cancer (Balkan countries, Serbia)	[49,50]
Reynosin Alantolactone Santamarine	*Aucklandia lappa* (Mu Xiang) *Inula helenium* (Xuan Fu Hua)	Anorexia, nausea and abdominal pain (Korea); Cancer, ulcers, and viral, bacterial and parasitic infections	[51,52,53]
**Alkaloids**			
*Isoquinoline alkaloids* Tetrahydropalmatine	*Stephania delavayi* *Stephania sinica* (Jin Bu Huan)	Components of Jin Bu Huan (TCM) used as a sedative, antispasmodic, analgesic and decongestant	[54,55,56]
*Quinazolinone alkaloids* Febrifugine	*Dichroa febrifuga* (Chang Shan)	Malaria	[57,58]
*Pyrrolizidine alkaloids* Senecionine Amabiline Intermedine Lycopsamine Supinine Retrorsine Senecionine Senkirkine	*Senecio jacobaea* *Senecio flaccidus* *Senecio scandens* *Senecio vulgaris* *Senecio asperum x officinale* *Heliotropium lasiocarpum* *Symphytum officinale* *Cynoglossum officinale* *Crotalaria verrucosa* *Holarrhena antidysenterica* *Cassia auriculata* *Borago officinalis* *Tussilago farfara*	Inflammatory conditions such as gout, arthritis, thrombophlebitis, skin wounds (bruises, fungal infections, ulcers, fractures, strains), varicose veins, oral lesions (gargle), bronchitis, allergies, gastritis, gastroduodenal ulcers, and diarrhea bronchitis, asthma, and cough; Gastrointestinal and urinary ailments and mainly for pulmonary complaints, blood purification, especially for rheumatism and skin irritations	[59,60,61,62,63,64,65,66,67,68,69,70,71]
*Sympathomimetic alkaloids* Ephedrine Pseudoephedrine Methylephedrine Norephedrine	*Ephedra sinica* (Ma Huang)	Bronchitis and asthma	[72,73,74]
*Piperidine alkaloids* Flavokavain A Flavokavain B	*Piper methysticum* (Kava)	Asthma, rheumatism, urological problems, menopausal symptoms, gonorrhea, vaginitis, nocturnal incontinence, weight loss, and insomnia	[4,5,75,76,77,78,79,80,81]
**Anthraquinones**			
*Free anthraquinones* Emodin Aloe-emodin Rhein Physcion	*Polygonum multiflorum* (He Shou Wu, Fo-Ti) *Polygonum cuspidatum* (Hu Zhang)	Tumor, bacterial infections, inflammatory diseases, HIV, renal insufficiency, diabetes mellitus, alopecia, atherosclerosis, hyperlipidemia, and neurodegenerative cardiovascular disease prevention	[82,83,84,85,86]
*Anthraquinone glycosides* Aloe-emodin-8-*O*-glucoside Emodin-8-*O*-glucoside Chrysophanol-8-*O*-glucoside Rhein-8-glucoside, Physcion-8-*O*-glucoside Emodin-1-*O*-glucoside	*Polygonum multiflorum* (He Shou Wu, Fo-Ti) *Polygonum cuspidatum* (Hu Zhang)	Tumor, bacterial infections, inflammatory diseases, HIV, renal insufficiency, diabetes mellitus, alopecia, atherosclerosis, hyperlipidemia, and neurodegenerative cardiovascular disease prevention	[87]
Sennosides	*Senna alexandrina* (Fan Xie Ye)	Constipation, gonorrhea, bronchial congestion, wounds, diarrhea, meteorism, skin diseases, dyspepsia, fever, and hemorrhoids	[88,89]
**Phenolic Acids**			
Phenolic acids and catechins	*Camellia sinensis*	Cancer and its prevention	[90,91,92]
Eugenol Methyleugenol Acetyleugenol	*Syzygium aromaticum* *Zingiber officinale* *Cinnamomum burmannii* *Foeniculum vulgare* *Piper betle* *Illicium verum* *Myristica fragnans*	Cancer, diabetes mellitus, and bacterial, inflammatory, fungal, protozoal and thrombotic diseases (many countries)	[93,94,95,96,97]
**Others**			
Nordihydroguaiaretic acid	*Larrea tridentata* (Chaparral)	Tuberculosis, arthritis, and cancer	[98]
Safrole	*Sassafras albidum* (Sassafras)	Topical anesthesia, muscle relaxation, blood purification, dental disinfectant, and skin disorders, hypertension, renal disorders, cancer, syphilis, and menstrual irregularity	[99,100,101]
α-Asarone	*Guatteria gaumeri* (Guatteria Gaumeri, Yumel)	Treatment of hypercholesterolemia and cholelithiasis (Mexico)	[102,103]

**Table 2:** Suspected nonphyto-hepatotoxins found in herbal medicinal products

Nonphyto-Hepatotoxins	Contaminated HMPs	Contamination Pathway	Toxic Effects	References
**Metals**				
Lead Mercury Arsenic Copper Cadmium Chromium Manganese Nickel Zinc Iron Selenium Antimony Thallium Tin	Sporadically detected in HMPs over the world	From soil, water, herbicides, pesticides or production processing	Oxidative stress; Lipid peroxidation; Lysosomal injury; Mitochondrial dysfunction; Hepatic DNA damage	[7,113,114,115,117,119]
**Mycotoxins**				
Aflatoxins (AF) Ochratoxin A (OTA) Fumonisins (FB) Zearalenone (ZEN) Deoxynivalenol (DON)	Ayurveda from India and traditional Chinese medicines, European and American herbal remedies, South Pacific medicinal plants (kava)	From *Aspergillus*, *Penicillium*, *Fusarium*, *Claviceps*, and *Alternaria* fungi during the storage conditions and processing of HMPs	Hepatic total antioxidant capacity decline; Lipid peroxidation increase; Potential hepatic apoptosis by upregulation of the apoptotic genes Caspase-3 and Bax	[78,120,121,122]
**Pesticidal and herbicidal residues**				
*Pesticides* Imazalil Thiacloprid Clothianidin Chlordane Dichlorodiphenyltrichloroethane (DDT) Lindane Pentachlorophenol Organophosphorus	Thoroughout the world	From agricultural activities; Water source and soil quality of cultivating place; From storage, transportation, covering contaminations	Hepatocellular hypertrophy and cytoplasm degeneration; Cholestasis; Decreased acetylcholinesterase activity; Hepatic mitochondrial toxicity	[114,123,124,125,126]
*Herbicides* Alachlor Acetochlor Metolachlor Quizalofop-p-ethyl Atrazine Paraquat dichloride	Thoroughout the world	From pollutants at cultivating place; From storage, transportation, covering contaminations	Hepatocellular enlargement and cytoplasm worsening; Decrease and obstruction of bile flow; Declined activity of liver acetylcholinesterase and hepatic mitochondrial dysfunction	[127,128,129,130,131]

## Abbreviations

CYP450	Cytochrome P450
DILI	Drug induced liver injury
GSH	Glutathione
HILI	Herb induced liver injury
HMP	Herbal medicinal product
HSOS	Hepatic sinusoidal obstruction syndrome
MP	Medicinal plant
PA	Pyrrolizidine alkaloid
RCT	Randomized controlled trial
ROS	Reactive oxygen species
RUCAM	Roussel Uclaf Causality Assessment Method
TCM	Traditional Chinese medicine
